# A novel insight into the antidepressant effect of cannabidiol: possible involvement of the 5-HT1A, CB1, GPR55, and PPARγ receptors

**DOI:** 10.1093/ijnp/pyae064

**Published:** 2024-12-06

**Authors:** Yang Miao, Fei Zhao, Wei Guan

**Affiliations:** Department of Pharmacology, The First People’s Hospital of Yancheng, Yancheng, Jiangsu, China; Department of Pharmacology, Jiangyin Hospital of Traditional Chinese Medicine, Jiangyin, Jiangsu, China; Department of Pharmacology, Pharmacy College, Nantong University, Nantong, Jiangsu, China

**Keywords:** depression, CBD, 5-HT1A receptor, neurogenesis, GPR55, inflammation

## Abstract

**Background:**

Depression is a prevalent and disabling disorder that poses serious problems in mental health care, and rapid antidepressants are novel treatments for this disorder. Cannabidiol (CBD), a nonintoxicating phytocannabinoid, is thought to have therapeutic potential due to its important neurological and anti-inflammatory properties. Despite major advances in pharmacotherapy in experimental animals, the exact mechanism of antidepressant-like effects remains to be elucidated.

**Methods:**

In this paper, we review the current state of knowledge on the antidepressant properties of CBD in numerous experimental and clinical studies.

**Results:**

Accumulating evidence suggests that CBD has antidepressant properties in humans and animals with few side effects, suggesting that CBD may be a potential antidepressant. Furthermore, we discuss that CBD may therefore provide a potential treatment to exert antidepressant-like effects through various molecular targets, reducing inflammation, and enhancing neurogenesis.

**Conclusions:**

Taken together with the growing popularity of CBD as a medicine, these findings extend the limited knowledge on the antidepressant effects of CBD. This potentially opens up new therapeutic means for the patients with depression.

## INTRODUCTION

Depression is one of the most common psychiatric disorders, characterized by depressed mood, social isolation, and anhedonia, which vary between patients.^1^ The World Health Organization estimates that over 322 million people of all ages suffer from depression, and it is the leading cause of disability worldwide.^2^ Depression can be characterized by a range of somatic, emotional, and behavioral symptoms, the most dangerous of which include persistent sad, anxious, or “empty” mood, and thoughts of death and suicide with multiple episodes, leading to a significant reduction in overall quality of life.^3^

Stress has long been recognized as a common etiological factor in the development of depression.^4^ Chronic social stress can cause structural and functional changes in the brains of patients with depression.^5^ Meanwhile, social stress induces increased levels of inflammation and impaired neuroprotection, which are associated with the pathogenesis of depression.^6,7^ In addition, a growing body of data suggests that various genetic and environmental factors contribute to the onset of depression.^8^ Remarkably, research shows that only about 20% to 25% of individuals suffer from depression after experiencing major stressful events,^9^ while large numbers of people successfully adapt to social stress and maintain healthy psychological and physiological functioning.^10^ This suggests that the development of depression in humans is closely linked to individual differences that determine either resilience or susceptibility.

It is now well recognized that neuroinflammation is a key factor that interacts with the other pathophysiology of depression, including dysfunction of monoamine neurotransmitter system, abnormalities of the hypothalamic-pituitary-adrenal axis, and defects in hippocampal neurogenesis.^11^ Some recent clinical and preclinical evidence suggests that some alterations in the community of microorganisms throughout the gastrointestinal tract (gut microbiota) are associated with depressive disorders.^12^ Although some progress has been made in understanding the pathological and pharmacological mechanisms of depression, the cellular and molecular etiology of depression remains largely unknown.

To date, the mainstay of treatment for depression has been pharmacological and psychological interventions. Psychological therapies are effective in treating most patients with mild to moderate depression, while only a small proportion of patients with a diagnosis of major depressive disorder (MDD) are referred to mental health services and require pharmacotherapy.^13^ Despite advances in pharmacological treatments, it is estimated that 10%-30% of patients are refractory to standard interventions,^14^ even after treatment with multiple medications. In recent years, ketamine has become an emerging treatment option due to its rapid and robust antidepressant effects.^15-18^ However, it has several adverse effects, including psychiatric, psychotomimetic, cardiovascular, neurological, and other side effects.^19^ Electroconvulsive therapy (ECT) has been extensively studied as another alternative and well-known treatment for treatment-resistant depression (TRD).^20^ However, ECT may cause cognitive impairment, delirium, musculoskeletal pain/injury, and anesthesia-related complications,^21,22^ which limits its use. Therefore, the development of novel and effective treatments for depression is very urgent and important.

In recent years, natural products and their biological activities with minimal or no side effects have gained widespread attention as therapeutic alternatives. Some compounds, mainly isolated from plants, have neuroprotective effects and are a valuable source for the development of new drugs for depression.^23^ It is therefore important to find safer and more effective antidepressant compounds from a wide range of natural products. The cannabis plant has been used for many centuries to treat a variety of ailments including appetite, anxiety, depression, sleep, and migraine.^24^ In most varieties of cannabis, the 2 most common cannabinoids are delta‐9‐tetrahydrocannabinol (THC) and cannabidiol (CBD), although over 100 phytocannabinoid compounds are produced by the cannabis plant.^25,26^ These compounds have different pharmacological effects. For example, THC is the main psychoactive component and exerts its psychoactive effects by engaging the endogenous cannabinoid (endocannabinoid) system.^27^ Although THC has received considerable attention for its appetite-stimulating and antinausea effects,^28^ exposure to high concentrations of THC could result in psychological events and adverse effects, such as psychiatric symptoms (anxiety, depression, and others), decreased level of consciousness, respiratory distress, and gastrointestinal reactions (nausea and vomiting),^29,30^ mainly associated with recreational use. In contrast, CBD is a phytocannabinoid with no psychotomimetic or other adverse effects and acts outside of the endocannabinoid system (ECS) due to its low binding affinity for these endogenous receptors.^31,32^ Recently, there has been increased interest in CBD, as several studies have shown promising neuroprotective efficacy without the potential for abuse or dependence and without the typical spectrum of side effects common after treatment with THC.^33,34^ In addition, there is growing evidence that CBD has antidepressant effects in experimental animal models of depression.^35^ Despite the observation of the previously reported antidepressant-like effects in animal models, the exact mechanism remains poorly understood. Therefore, this paper reviews the molecular targets, pharmacokinetics (PK), and safety of CBD. Additionally, the existing evidence for its potential therapeutic effects in depression is described. However, as CBD gains popularity and its use for diseases expands worldwide, the adverse effects and toxicity of CBD remain largely unknown. Therefore, a comprehensive and detailed review is warranted to provide insight into this topic.

## OVERVIEW OF CBD

**Figure 1.**
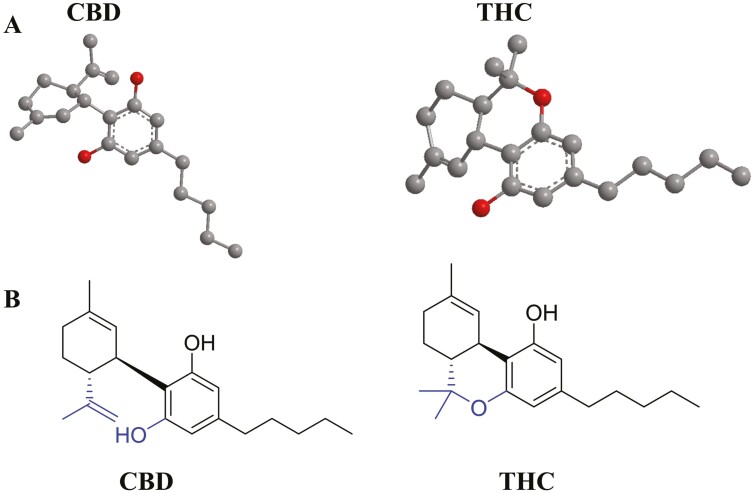
(A) 3D chemical structures of cannabidiol (CBD) and Δ9-tetrahydrocannabinol (THC). Gray spheres represent carbon atoms, and red spheres represent oxygen atoms.^40^ (B) Chemical structures of THC and CBD. Figure is redrawn from Castillo-Arellano et al.^41^

*Cannabis sativa* (Linneo, 1753), the herbaceous plant, has been known and used for thousands of years as a medicine and for recreational and spiritual purposes.^36,37^ It is the most commonly used addictive substance after alcohol, caffeine, and tobacco.^38^ Approximately 188 million people worldwide use cannabis annually, with the estimated number of cannabis users worldwide increasing by approximately 16% between 2006 and 2016.^39^  *Cannabis sativa* is a plant composed of hundreds of constituents, including more than 100 cannabinoids, the most common of which are THC and CBD (Figure 1). THC was discovered in 1964 and is recognized as the primary psychoactive compound that drives the abuse potential of cannabis.^31^ CBD, the second most abundant active ingredient in the cannabis plant, is a nonintoxicating component that does not convert to THC in the human body.^33^ CBD is a cannabinoid that was first isolated from Mexican marijuana (*Cannabis sativa* L.) by Adams, Hunt, and Clark in 1940.^42^ Shortly afterward, the structure of CBD from Lebanese hashish was determined by Mechoulam and Shvo in 1963.^43^ CBD is a 21-carbon terpenophenolic compound with a molecular weight of 314.464 g/mol.^44^ It is arranged into a cyclohexene ring, a phenolic ring, and a pentyl side chain, while the terpenic and phenolic rings are located in planes almost perpendicular to each other.^45^ In addition, the saturated exocyclic C-C double bond structurally prevents the conversion of CBD to the psychoactive THC. Current research shows that CBD possesses broad pharmacological activities, including antioxidant, anti-inflammatory, immunomodulatory, neuroprotective, anticancer, antinociceptive, and analgesic properties.^46^ Despite the broad spectrum of pharmacological effects, evidence for CBD’s therapeutic promise is largely based on preclinical cellular and rodent studies, and only few controlled clinical trials have been conducted to investigate its therapeutic potential. There has been an incredible amount of media coverage on the health benefits of CBD, and numerous CBD products are available. Epidiolex®, a purified form of oral CBD solution,^47^ was approved by the US Food and Drug Administration (FDA) in 2018 as an adjunctive treatment for 2 rare epilepsies: Lennox-Gastaut syndrome and Dravet syndrome in pediatric patients aged 1 year and older.^48^ However, non-FDA-approved CBD products may contain sufficient THC or pesticide or heavy metal contamination that the specific effects of CBD in the products cannot be determined.^49^

## PHARMACOKINETICS

CBD is a highly lipophilic molecule and is therefore rapidly distributed to the brain, adipose tissue, and other organs.^50,51^ In clinical practice, it is usually administered orally.^52^ In recent years, several pharmacokinetic studies have been conducted on the prescription CBD product Epidiolex®. Although numerous studies have investigated the PK of CBD-THC combinations (Table 1),^71^ the kinetic behavior of CBD alone is not yet fully elucidated.

**Table 1.** Human studies reporting pharmacokinetic (PK) parameters for CBD.

Population (Total *n*)	Administration	CBD dose	Samples	PK details T_max_ C_max_ t_1/2_	References
Healthy adults w/a history of cannabis use (*n* = 12)	Sublingual	20 mg (GW-3009–01)	Plasma	2.17 h 2.05 ng/mL ------	^53^
Healthy male and female subjects (*n* = 12)	Oromucosal spray sublingual Oromucosal spray buccal Oromucosal spray oro-pharyngeal	10 mg (GW pharmaceuticals)	Plasma	1.63 h 2.5 ng/mL 1.44 h 2.79 h 3.02 ng/mL 1.81 h 2.04 h 2.61 ng/mL 1.76 h	^54^
Normal healthy male volunteers (*n* = 24)	Oromucosal spray sublingual	10 mg (GW pharmaceuticals)	Plasma	4.22 h 3.33 ng/mL 1.81 h	^55^
Adult polydrug users (*n* = 41)	Oral solution	750 mg 1500 mg 4500 mg (Epidiolex®)	Plasma	5.11 h 336.2 ng/mL ------ 6.13 h 524.5 ng/mL 4.07 h 426.9 ng/mL	^56^
Adult regular cannabis users (*n* = 8)	Oral	800 mg (STI Pharmaceuticals)	Plasma	3 (2-6) h 77.9 (1.6-271.9) ng/mL -----	^57^
Adults w/refractory epilepsy (*n* = 8)	Oral	200-300 mg (Viero Health Violet Formulation)	Plasma	Fed: 2.4 (1-6) h 0.45 (ng/mL)/mg 24.3 h Fasted:3.2 (2-5) h 0.03 (ng/mL)/mg 38.9 h	^58^
Huntington’s patients (*n* = 14)	Oral	10 mg/kg daily for 6 weeks (National Institute on Drug Abuse)	Plasma	41.4-113 h 68.2 h	^59^
Patients aged 4-10 years with Dravet syndrome (*n* = 34)	Oral solution	5, 10, 20 mg/kg twice daily for 22 days (Epidiolex®)	Plasma	2.5 h 29.3-37.6 ng/mL	^60^
Adult men w/a history of cannabis use (*n* = 5)	Intravenous Smoking	20 mg 19 mg	Plasma	Mean: 686 SD: 239 Mean: 24 h Mean: 110 SD: 55 Mean: 31 h	^61^
Healthy adults (*n* = 6)	Oral solution	Single ascending dose: 1500, 3000, 4500, 6000 mg (Epidiolex®)	Plasma	3-5 h 292.4 ng/mL 14.43 h 533.0 ng/mL 14.39 h 722.1 ng/mL 16.61 h 782.0 ng/mL 15.42 h	^62^
Healthy adults (*n* = 9)	Oral solution	Multiple dose: 750, 1500 mg twice daily for 6 days (Epidiolex®)	Plasma	3 (2.5-5) h 330.3 ng/mL 56.41 h (day 7) 3 (2-4) h 541.2 ng/mL 60.54 h	^62^
Healthy adults (*n* = 12)	Oral solution	1500 mg	Plasma	Fasted: 3.5 (2.5-5.03) h 335.4 ng/mL 30.33 h Fed: 3 (1.5-5) h 1628 ng/mL 24.40 h	^62^
Subjects with mild to severe hepatic impairment (*n* = 8 each for mild and moderate, *n* = 6 for severe) relative to matched subjects with normal hepatic function (*n* = 8)	Oral solution	200 mg (Epidiolex®)	Plasma	2.8 (1.5-5.0) h 233 ng/mL 15.7 h 2.0 (1.5-3.0) h 354 ng/mL 20.5 h 2.5 (2.0-5.0) h 381 ng/mL 22.1 h 2.3 (1.5-5.0) h 148 ng/mL 8.58 h	^63^
Pediatric patients (aged 1 to ≤ 17 years) with treatment-resistant epilepsy (cohort 1 [10 mg/kg/day], *n* = 20; cohort 2 [20 mg/kg/day], *n* = 20, and cohort 3 [40 mg/kg/day], *n* = 21)	Oral solution	A single dose (5 mg/kg, 10 mg/kg, or 20 mg/kg, respectively) on day 1, and no drug was given on days 2 and 3, 5 mg/kg, 10 mg/kg, or 20 mg/kg twice daily (10 mg/kg/day, 20 mg/kg/day, or 40 mg/kg/day, respectively) from day 4 to day 10	Plasma	Day1: 2.6 (1.0-8.0) h 59.03 ng/mL 31.3 h 4.0 (1.0-8.1) h 110.5 ng/mL 33.5 h 3.2 (1.0-24.0) h 256.9 ng/mL 21.6 h Day10: 3.0 (1.0-4.2) h 119.6 ng/mL 2.0 (0.0-6.0) h 220.0 ng/mL 3.0 (0.0-6.0) h 426.8 ng/mL	^64^
24 volunteers (12 male and 12 female, age 18-45 years)	Oral capsule	5.4 mg once a week for 3 weeks (Scherer GmbH & Co. KG)	Plasma	0.99 (0.5-2) h 0.93(0-2.6) ng/mL	^65^
Cannabis smokers (*n* = 9)	Oromucosal spray	5, 15 mg (GW pharmaceuticals)	Plasma	3.6 (1.0-5.5) h Mean (SE):1.6 (0.4) 4.6 (1.2-5.6) hMean (SE): 6.7 (2.0)	^66^
Cannabis smokers (*n* = 10)	Cannabis cigarette	2 mg (National Institute on Drug Abuse)	Plasma	0.25 h0.03 ng/mL	^67^
Healthy adults (male, *n* = 15)	A PTL401 capsule (self-emulsifying oral drug delivery system)	10 mg (STI pharmaceuticals)	Plasma	1.25(0.5-4.0) h 2.94 ng/mL 3.21 h	^68^
Healthy adults (male, *n* = 9)	Oromucosal spray	10 mg (Sativex®)	Plasma	3 (1-5) h 0.5 ng/mL	^69^
Healthy adults (male, *n* = 24)	Oromucosal spray	5, 10, 20 mg (GW pharmaceuticals)	Plasma	1.00 (0.75-1.50) h 0.39 ng/mL 5.28 h 1.39 (0.75-2.25) h 1.15 ng/mL 6.39 h 1.00 (0.75-1.75) h 2.17 ng/mL 9.36h	^70^
Healthy adults (male, *n* = 24)	Oromucosal spray	5, 10, 20 mg 9 days (GW pharmaceuticals)	Plasma	1.64 (1.00-4.02) h 0.49 ng/mL 1.27 (0.75-2.52) h 1.14 ng/mL 2.00 (1.02-6.00) h 3.22 ng/mL	^70^

Abbreviation: CBD, cannabidiol.

As mentioned above, oral administration is the primary route used in controlled human studies, with doses ranging from 20 to 6000 mg.^53,62^ The PKs of CBD are complex, and the bioavailability of oral CBD is low in different species (varying between approximately 6% and 19%).^51,72,73^ For example, the oral bioavailability of CBD in humans is approximately 6% under fasting conditions or highest (19%) when consumed with a high-fat meal.^74,75^ Therefore, it may be advisable to administer CBD orally in a fed state to allow for optimal absorption. We speculate that the lower oral bioavailability of CBD is likely due to its highly lipophilic nature, resulting in poor gastric solubility. Therefore, when CBD is administered in capsules, its low water solubility and absorption lead to variable PK, whereas CBD administered in the fat content of food or other routes of administration such as oral-mucosal/sublingual delivery through sprays or lozenges has less variability. CBD is extensively metabolized in the liver and mainly hydroxylated to 7-OH-CBD and 7-COOH-CBD by cytochrome P450 (CYP) 2C19 and to a lesser extent by CYP3A4.^76^ More than 84% of CBD is excreted in the feces and approximately 8% is excreted in the urine.

Taylor and colleagues conducted a study to assess the safety, tolerability, and PK of CBD oral solution (Epidiolex in the United States) in healthy adult volunteers.^62^ They found that CBD appeared rapidly in subjects’ plasma after single oral doses (1500, 3000, 4500, or 6000 mg under fasting conditions), with a time to maximum plasma concentration (*t*_max_) of approximately 4-5 h,^62^ which was independent of dose. In summary, CBD was generally well tolerated, and all adverse events (AEs) were of mild or moderate severity, with none being severe or serious.

Millar et al. performed a systematic search of PubMed and excerpta medica database (EMBASE) to retrieve all articles reporting PK data for CBD in humans.^71^ The mean half-life (*t*_½_) of CBD was reported to be 2-5 days after chronic oral administration,^59^ 1.44-10.86 h after oromucosal spray (5-20 mg),^66,77,78^ 24 h after intravenous administration,^61^ and 31 h after smoking.^61^

In summary, these findings demonstrate that despite its widespread use in humans, the PK of CBD varies markedly depending on the route of administration and dosing regimen. Furthermore, changes in the route of administration, such as sprays, an aerosol, and a nebulizer, increase bioavailability. In addition, plasma levels of CBD were increased when CBD was dissolved in the fat content of food, as demonstrated by Cherniakov et al. Co-administration of a pro-nanoliposphere formulation with oral CBD increased systemic availability by approximately 6-fold.^79^ Despite this evidence suggesting a favorable safety profile for CBD, there are several notable concerns regarding AEs associated with the use of CBD, and further work is warranted.

## RECEPTOR BINDING PROFILE OF CBD

**Figure 2.**
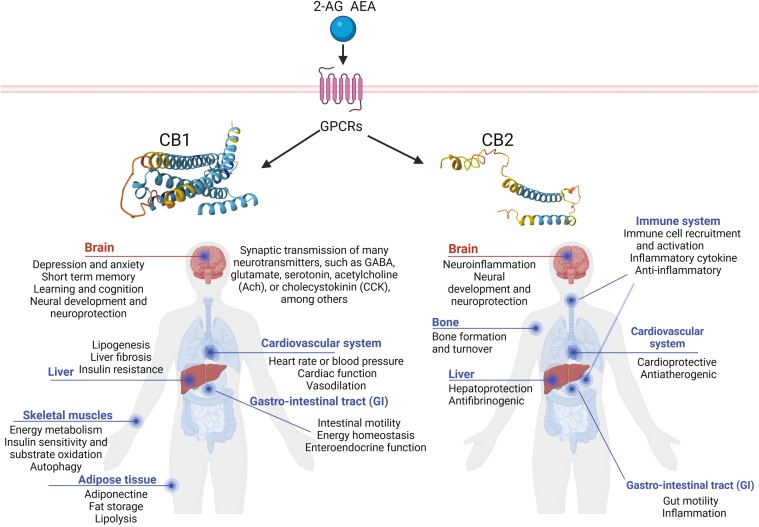
Major localization sites and associated functions of the CB1R and CB2R in the human body. Both CB1 and CB2 cannabinoid receptors are GPCRs that primarily couple to G proteins of the Gi and Go classes. The majority of CB1Rs expressed in the human body are found in the brain, including the olfactory bulb, hippocampus, basal ganglia, cerebellum, cerebral cortex, septum, amygdala, and hypothalamus, where they are involved in various neurological activities. In addition, CB1R is also abundantly expressed in the peripheral nervous system (PNS) and peripheral tissues in a region-specific manner. CB2R is mainly found in immune cells such as macrophages, B lymphocytes, and blood stem cells, and in immune organs such as the spleen, tonsils, and thymus, and to a lesser extent in the cerebral cortex, cerebellum, and the gastrointestinal tract. The crystal structures of CB1R and CB2R were generated using the AlphaFold Monomer v2.0 pipeline. The figure was generated using BioRender (Agreement number: OR27NE69ML). CB1R, CB1 receptor; GPCR, G-coupled protein receptors.

The ECS is a complex molecular/biological system discovered in 1988 by scientists Allyn Howlett and W.A. Devane.^80,81^ The ECS is primarily responsible for maintaining homeostasis and a balance in metabolism and energy. In addition to regulating physiological processes, the ECS directly influences brain plasticity, learning and memory, neural development, and the regulation of stress and emotions (Figure 2).^82^ The ECS is composed of cannabinoid receptors (CBRs), including G-coupled protein receptors (GPCRs) (eg, cannabinoid CB1 receptor [CB1R] and cannabinoid CB2 receptor [CB2R]), ligand-sensitive ion channels (eg, transient receptor potential vanilloid 1 [TRPV1]), and nuclear receptors (eg, peroxisome proliferator-activated receptors [PPARs]); the endogenous ligands arachidonoylethanolamide (anandamide) or N-arachidonoylethanolamine (AEA) and 2-arachidonoylglycerol (2-AG); specific proteins involved in the endocannabinoid biosynthesis and degradation enzymes such as fatty acid amide hydrolase (FAAH).^83^ The best-studied components of the ECS in mammalian systems are the 2 CB1Rs and CB2Rs and their major endocannabinoid ligands, AEA and 2-AG.

### Cannabinoid Receptors

CB1R and CB2R are GPCR proteins with 7 transmembrane-spanning domains, originally discovered in the membranes of neuronal cells and macrophages, respectively.^84,85^ As members of the GPCR family, CB1R is encoded by the gene *CNR1* and consists of 472 amino acids in humans (473 amino acids in rats and mice, with 97%-99% amino acid sequence identity between these species),^86^ while CB2R is encoded by the gene *CNR2* with 360 amino acids in humans, which shares only 44% sequence homology with CB1R at the protein level and is generally coupled to the activation of heterotrimeric G proteins of the G_i_ and G_o_ classes.

### N-Arachidonoylethanolamine and 2-Arachidonoylglycerol

**Figure 3.**
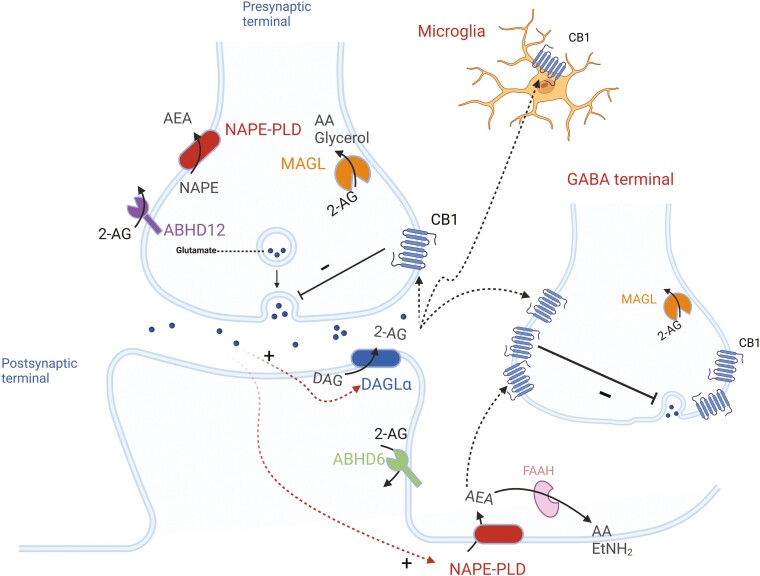
Simplified scheme of endocannabinoid retrograde signaling mediated synaptic transmission. The endocannabinoid system (ECS) is now known to exist in almost all animal phyla and consists of 2 known cannabinoid receptors (CB1 and CB2), 2 known endocannabinoids, anandamide or AEA and 2-AG, and their metabolic enzymes. AEA is catalyzed from N-acyl-phosphatidylethanolamine (NAPE) by NAPE-specific phospholipase D (NAPE-PLD) or by other routes not involving NAPE-PLD and is catabolized mainly by the fatty acid amide hydrolase (FAAH). FAAH is located primarily in postsynaptic terminals and is responsible for the degradation of AEA to arachidonic acid (AA) and ethanolamine (EtNH2). 2-AG is biosynthesized from diacylglycerol (DAG) by diacylglycerol lipase alpha (DAGLα). The degradation of 2-AG is mainly by monoacylglycerol lipase (MAGL) into AA and glycerol, and to a lesser extent by the alpha/beta domain-containing hydrolase 6 (ABHD6) and alpha/beta domain-containing hydrolase 12 (ABHD12). As lipids, endocannabinoids, mainly 2-AG, readily cross the membrane and travel in a retrograde fashion to activate CB1Rs located in the presynaptic terminals. Activated CB1Rs will then inhibit neurotransmitter (NT) release by suppressing calcium influx. 2-AG can also activate CB1Rs located in astrocytes, leading to the release of glutamate. Blunted arrow indicates inhibition; thick arrows indicate enzymatic process; dummy arrows indicate translocation. AEA, N-arachidonoylethanolamine; 2-AG, 2-arachidonoylglycerol; GABA, gamma-aminobutyric acid. The figure was adapted from Seillier et al.^92^ and generated using BioRender (Agreement number: SP27NE5SL2).

Meanwhile, AEA and 2-AG, as derivatives of arachidonic acid, have been discovered to act as endogenous agonists of CBRs.^87^ 2-AG acts as a full agonist at both CBRs with moderate to low affinity, whereas AEA is a high-affinity, partial agonist of CB1R, and almost inactive at CB2R.^88^ In addition, AEA is a potent partial agonist at the G protein coupled receptor 55 (GPR55) receptor and a low-affinity full agonist at the TRPV1 calcium channel, whereas 2-AG is more potent than AEA at the CB1, CB2, and GPR55 receptors. Despite similarities in chemical structure, AEA and 2-AG are synthesized, transported, and inactivated differently in their respective target tissues. Specifically, AEA is catalyzed from N-acylphosphatidylethanolamine (NAPE) by NAPE-specific phospholipase D (NAPE-PLD) or by other routes not involving NAPE-PLD and is catabolized mainly by FAAH.^89^ Diacylglycerol lipase α and β, the main biosynthetic enzymes of 2-AG, are highly expressed in the nervous system and immune systems, respectively.^90,91^ The degradation of 2-AG is primarily by monoacylglycerol lipase and to a lesser extent by alpha/beta domain-containing hydrolase 6 and alpha/beta domain-containing hydrolase 12 (Figure 3).^93^

### Transient Receptor Potential Vanilloid 1

**Figure 4.**
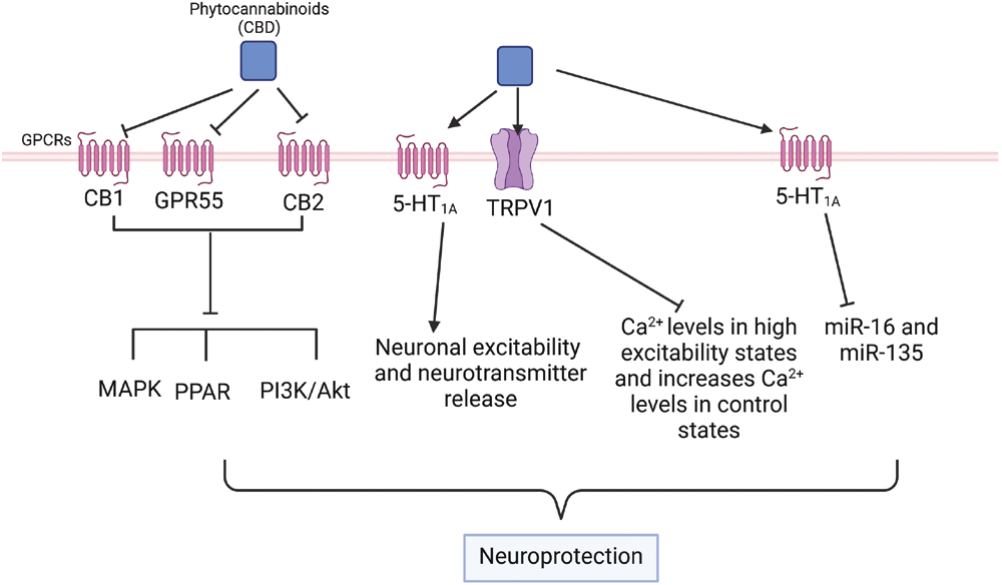
Multiple molecular targets for CBD. CBD has multiple molecular targets within the cell. It acts as an antagonist for CB1 and CB2 receptors and also appears to function as a GPR55 antagonist. In contrast, many of CBD’s effects are mediated through activation of serotonin receptor subtype 1A (5-HT1A) receptors in the central and peripheral nervous system, which regulate neuronal excitability and neurotransmitter release. In addition, a proposed molecular target for CBD is the TRPV1 receptor, and when activated by CBD, TRPV1 causes an increase in intracellular Ca^2+^. Furthermore, using mitochondrion‐specific Ca^2+^ sensors (Rhod‐FF, AM), CBD has been shown to reduce [Ca^2+^]_i_ levels in high excitability states and increase [Ca^2+^]_i_ levels in control states in primary cultured hippocampal cells.^96^ In addition, the therapeutic properties of CBD may involve epigenetic mechanisms, including DNA methylation, histone modifications, and the regulation of miRNA expression.^97^ For example, the antidepressant effects of CBD in a rat model of depression were associated with changes in miR-16 and miR-135 in the vmPFC and were mediated by the 5-HT1A receptor.^35^ The figure was generated using BioRender (Agreement number: IG27NE5X92). CBD, cannabidiol; TRPV1, transient receptor potential vanilloid 1; vmPFC, ventromedial prefrontal cortex.

Research suggests that many cannabinoids, including THC, exert their physiological and pharmacological effects by engaging the ECS,^94^ whereas CBD has a low binding affinity for these endogenous receptors (CB1 and CB2) and instead acts outside of the ECS.^95^ Although the direct effects of CBD on CBRs appear limited, over 65 molecular targets for CBD have been identified, including the TRPV1 channel, GPR55, peroxisome proliferator-activated receptor-γ (PPARγ), serotonin receptor subtype 1A (5-HT1A), dopamine receptor (D2), and the equilibrative nucleoside transporter 1, which have been proposed as plausible targets of CBD (Figure 4).^95,98,99^

For example, CBD is a low-potency, full agonist for TRPV1 and causes rapid desensitization of TRPV1.^100,101^ TRPV1 is a Ca(2+)-permeable, nonselective cation channel that is activated by noxious stimuli, heat, protons (pH < 5.9), and various, mostly noxious, natural products such as capsaicin.^102^ Under neuroinflammatory conditions, there is a marked increase in the sensitivity and density of TRPV1, while CBD binding to TRPV1 promotes the desensitization of this channel and consequently reduces neuroinflammation.^103^ In addition, Iannotti et al. showed that CBD activated and rapidly desensitized the TRPV1 channel and reduced neuronal hyperexcitability in rat hippocampal tissue using patch-clamp analysis.^104^

### Serotonin Receptor Subtype 1A

CBD is also an agonist at 5-HT1A receptors both in vitro and in vivo.^105,106^ The finding that CBD could act as a 5-HT1A receptor agonist was first demonstrated by Russo et al.^105^ They observed that CBD displaced 8-OH-DPAT (a 5-HT1A receptor agonist) from the cloned human 5-HT1A receptor in a concentration-dependent manner. In addition, administration of CBD into the ventral medial prefrontal cortex (vmPFC) of rats induced antidepressant-like effects, possibly through indirect activation of the 5-HT1A receptor, while a 5-HT1A receptor antagonist, WAY100635, blocked CBD-induced antidepressant-like effects,^107^ suggesting the involvement of 5-HT1A receptors in the effects of CBD in vivo.

### GPR55

Recently, studies have implicated GPR55 in the mechanisms of action of CBD.^108^ Evidence from Zhang et al. showed that treatment with CBD alleviated neurobehavioral abnormalities in maternal immune activation (MIA) mice by inhibiting the lipid lysophosphatidylinositol (LPI)-GPR55 signaling pathway, and a GPR55 antagonist (CID16020046) mimicked the effect of CBD on MIA mice and alleviated depression- and anxiety-like behaviors in MIA offspring.^109^

In summary, these findings indicate that CBD has a nonselective receptor binding profile, which may be due to the multiplicity of CBD molecular targets. As the involvement of these receptors is only partial, further in vivo studies are clearly needed to investigate their possible involvement in CBD’s behavioral effects.

## CBD AND DEPRESSION

### Clinical Studies

Depression is a common, persistent psychiatric condition characterized by depressed mood, poor sleep, low appetite, poor concentration, and lack of energy that co-exist with other chronic illnesses. Clinical studies have confirmed that CBD has antidepressant properties. Wieckiewicz et al. conducted a questionnaire and reported that the majority of the respondents (53%) claimed that CBD made them feel better overall and 88% of the respondents would be more likely to take CBD than a prescription drug from a psychiatrist.^110^ They further stated that their study was exploratory in nature, and they did not pose any specific research questions. In another study, Rapin et al. used the Edmonton Symptom Assessment Scale-revised version to evaluate the effect of CBD on patients with depression in a medical cannabis clinical setting in Canada. They found that CBD-rich treatment had a beneficial effect on depression symptoms in patients with moderate to severe symptoms,^111^ suggesting that the overall efficacy of CBD treatment is primarily in patients with moderate to severe symptoms. In addition, Martin et al. conducted a longitudinal web-based survey study and assessed symptoms of depression in a convenience sample of medical cannabis users with depression compared to a non-using control group.^112^ The use of CBD-dominant cannabis products was found to reduce depressive symptoms in clinically depressed populations.^112^ Further investigation revealed that no differences in the incidence of AEs were observed between CBD and non-CBD users,^112^ suggesting that CBD is a safe and well-tolerated medication for patients with depression. However, the authors also identified several limitations in their study, for example, these results in the study relied entirely on participant self-report. In addition, the survey was conducted over the internet, which limited the ability to rule out respondent error in completing the survey. Also, a small group of respondents is not necessarily representative of the population. Much more research from well-designed clinical trials is therefore needed to support its widespread use as a treatment for depression.

In a randomized, double-blind, placebo-controlled trial, Berger et al. found that CBD could reduce the severity of depression and had an adequate safety profile in young people with TRD disorders.^113^ The study did not evaluate the longer term safety of CBD. These results are consistent with those previously reported by Laczkovics et al. who showed that administration of CBD capsules at different doses (starting dose 100 mg up to 600 mg over 8 weeks) attenuated depressive symptoms in a 16.9-year-old patient with multiple substance use disorder (cannabis, MDMA, cocaine, ecstasy) and major depression.^114^ They also found that the patient did not report any side effects.^114^ Taken together, these findings suggest that CBD can reduce depressive symptoms in patients and that it has demonstrated safety and a good side-effect profile in many clinical trials.

Confirming and extending these findings, O’Neill et al. showed that CBD attenuated dysfunction in the mediotemporal and prefrontal regions of psychosis patients by using the functional magnetic resonance imaging paradigm, such that activation under its influence was intermediate between the placebo condition and healthy controls.^115^ They also confirmed that CBD produced trend-level symptom reduction in psychosis patients via attenuation of hippocampal-striatal functional connectivity.^115^ In this context, it is worth emphasizing that normalization of mediotemporal-striatal functional connectivity may underlie the antipsychotic effects of CBD.

Nevertheless, the fact remains that CBD may exacerbate psychotic symptoms, and have a negative impact on the course of the illness. For example, in a systematic review of prospective controlled trials, Stanciu and colleagues found insufficient evidence for the efficacy of CBD in the treatment of affective and anxiety disorders.^116^ They also concluded that medical CBD should not be recommended for the treatment of patients with these disorders. In another randomized, double-blind, cross-over, placebo-controlled study, Martin-Santos et al. confirmed that there were no differences between CBD and placebo on any symptomatic, physiological variable in healthy volunteers.^117^ In line with these observations, Pinto et al. conducted a randomized, double-blind, placebo-controlled pilot study to assess the efficacy of adjunctive CBD in bipolar depression in a total of 35 participants.^118^ In this study, the authors found no significant difference in Montgomery-Åsberg Depression Rating Scale scores between the placebo and CBD groups.^118^ Notably, the same researchers also found that there were no other significant effects on the secondary outcomes.^118^ These data suggest that there is not enough research to support the evidence for the efficacy of CBD on depression. We believe that the conflicting reports on the effects of CBD on patients with depression may be partly explained by the different experimental protocols and sample sizes used by the investigators. To date, despite the strong evidence for the antidepressant therapeutic properties of CBD in patients, the mechanisms of action of CBD remain unclear and are likely to be diverse. Further clinical trials would be needed to investigate the mechanism of CBD’s antidepressant effects in patients.

### Preclinical Studies (Animal Models)

CBD is the most abundant nonpsychoactive component of cannabis plants, constituting up to 40% of the extract. Its beneficial pharmacological effects, including anti-inflammatory and antioxidant properties, have been extensively studied.^119^ In addition, the therapeutic potential of CBD has been evaluated in diabetes and its complications, acute and chronic pain, cardiovascular disease, cancer, and epilepsy,^120^ which are usually accompanied by oxidative stress and inflammation. Due to its nonpsychoactive properties and high human tolerability, recent discoveries of its antidepressant properties have attracted considerable attention. Among them, we cite those of Sartim et al. who pointed out that CBD administration in the rat vmPFC, either in the infralimbic (IL) or prelimbic (PL) subregions, reduced the immobility time in the forced swimming test (FST), an antidepressant-like effect.^107^ Consistent with these findings, Zanelati et al. reported that CBD treatment reduced immobility time in the FST in male Swiss mice, and its antidepressant-like effects were comparable to those of imipramine.^121^ Therefore, these findings raise the possibility that CBD may be useful in the treatment of psychiatric disorders thought to involve impairment of stress coping mechanisms, such as depression. Despite this body of evidence, data on the benefits of CBD in reducing the severity of depressive symptoms in patients are limited but promising. Here, we review the key biological effects of CBD and focus on the antidepressant properties of CBD in preclinical studies to better understand the therapeutic potential of CBD for depression.

#### Antidepressant-Like Effects of CBD in Animal Models of Depression: Possible Involvement of Serotonin 1A (5-HT1A) Receptors

Although activation of 5-HT1A receptors has been consistently implicated in the therapeutic effects of antidepressants,^122^ a link between these receptors and the antidepressant-like effects of CBD has not been investigated. Therefore, the aim of this review was to highlight the evidence that CBD would induce antidepressant-like effects in rodents subjected to chronic stress.

**Figure 5.**
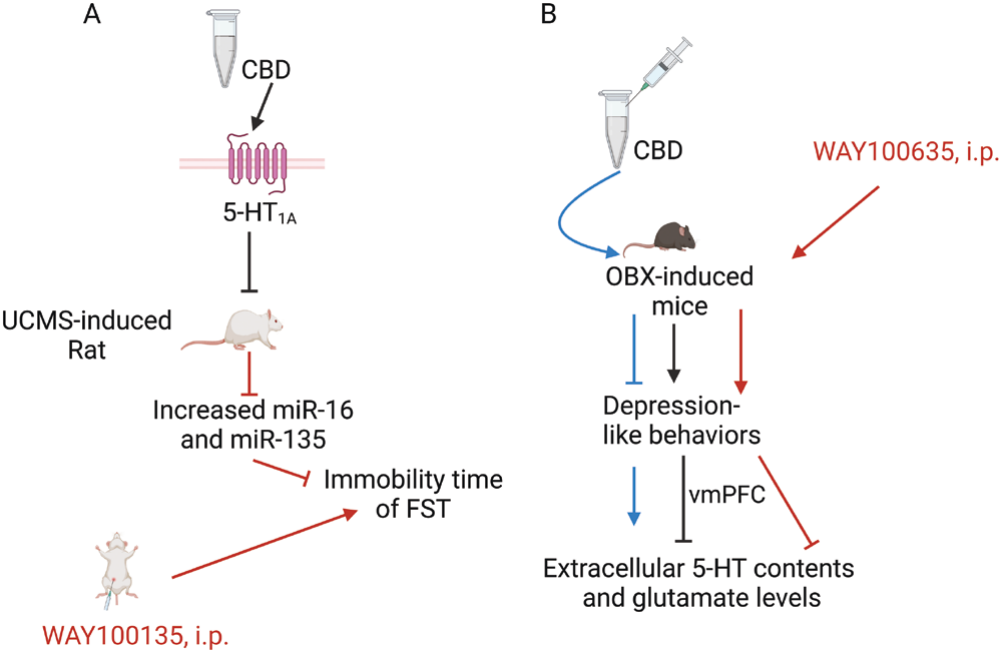
Serotonin receptor subtype 1A (5-HT1A) receptor mediates CBD-induced antidepressant-like effects. (A) CBD reversed the increased expression of miR-16 and miR-135 and the decrease in the 5-HT1A receptor gene (HTR1A) in the ventromedial prefrontal cortex (vmPFC) in rats subjected to UCMS, while WAY100135 (the 5-HT1A receptor antagonist) blocked the antidepressant-like effect of CBD on immobility time in rats.^35^ The UCMS is one of the most widely used, robust, and translatable models to study the neurobiological basis of major depression.^123^ (B) Acute CBD administration significantly increased extracellular 5-HT and glutamate levels in the vmPFC of OBX mice, whereas WAY100635 (0.3 mg/kg; i.p.) prevented the reversal of CBD-induced hyperactivity in OBX mice,^124^ suggesting that the rapid antidepressant-like effects of CBD act via the enhancement of serotonergic and glutamatergic transmission through the modulation of 5-HT1A receptors.^124^ The figure was generated using BioRender (Agreement number: DM27NE62NP). CBD, cannabidiol; OBX, olfactory bulbectomy; UCMS, unpredictable chronic mild stress.

A recent study by Bright et al. showed that CBD (10 mg/kg) reduced unpredictable chronic mild stress (UCMS)-induced increases in immobility time in male rats,^35^ indicating CBD has antidepressant effects in a rat model of depression (Figure 5). Importantly, CBD reversed the increased expression of miR-16 and miR-135 and the decrease in the 5-HT1A receptor gene in the ventromedial prefrontal cortex (vmPFC) in rats subjected to UCMS.^35^ Previous studies showed that miR-16 and miR-135 could be used as biomarkers for the diagnosis of depression.^125^

Notably, the same researchers conducted a pharmacological approach to show that WAY100135 (the 5-HT1A receptor antagonist) blocked the antidepressant-like effect of CBD on immobility time in rats.^35^ These results indicate that the antidepressant effects of CBD in a rat model of depression are associated with the downregulation of miR-16 and miR-135 in the vmPFC and are mediated by the 5-HT1A receptor. However, these findings are inconsistent with previous in vivo studies in humans, which reported that serum levels of miR-16 and miR-135 were significantly downregulated in patients with depression when compared to normal individuals.^125^ Similarly, cerebrospinal fluid miR-16 in MDD patients was significantly lower in patients with MDD than in controls, while blood miR-16 was not significantly different between the 2 groups.^126^ These conflicting results may be due to different tissue samples and individual differences, and further studies would be needed to investigate the possible mechanism of miR-16 in the physiopathology of depression.

In another study, Zanelati et al. evaluated the hypothesis that CBD would have antidepressant-like activity in mice using the FST.^121^ They found that CBD treatment (30 mg/kg) reduced immobility time in the FST in mice, while pretreatment with WAY100635 blocked the CBD-induced effect in the FST,^121^ suggesting that the antidepressant-like effects of CBD are probably mediated by activation of 5-HT1A receptors. In addition, they also investigated that CBD treatment did not alter hippocampal BDNF levels in mice.^121^ Despite these findings, the involvement of BDNF in the antidepressant behavioral effects of CBD cannot be ruled out, as several studies have shown that acutely increased BDNF levels were observed in both the hippocampus and PFC of mice.^127^ We suggest that differences in the age and species used or CBD dose (30 vs 10 mg/kg) may have contributed to the different results.^121,127^

Linge et al. investigated the rapid antidepressant-like effects of CBD in bulbectomised mice (Figure 5).^124^ They showed that both acute (50 mg/kg, 30 min after administration; intraperitoneally [i.p.]) and chronic administration of CBD reduced the olfactory bulbectomy (OBX)-induced hyperactivity and decreased central activity in mice.^124^ In addition, chronic CBD reversed OBX-induced anhedonia after 7 days of administration.^124^ In vivo microdialysis studies showed that acute CBD administration caused a significant increase in extracellular 5-HT levels and glutamate levels in the vmPFC of OBX mice, while chronic CBD did not cause a significant change in absolute basal 5-HT levels, although it increased absolute basal glutamate levels in OBX-treated mice.^124^ Finally, WAY100635 (0.3 mg/kg; i.p.) prevented the reversal of OBX hyperactivity and increase in central activity induced by CBD (50 mg/kg) in OBX-treated mice,^124^ suggesting that the rapid antidepressant-like effects of CBD act via the enhancement of serotonergic and glutamatergic transmission through the modulation of 5-HT1A receptors. OBX, an animal model of depression with comorbid anxiety, has been extensively used for preclinical research in rodents,^128^ as it induces some behavioral, neurochemical, neuroendocrine, and immunological changes similar to those observed in depressed patients.^129,130^ However, in this study, the authors did not find an anxiolytic-like effect of CBD in sham animals,^124^ while a previous study showed acute administration of CBD in rats induced anxiolytic-like effects per se.^131^ This discrepancy could be due to methodological differences related to the use of different species and behavioral tests to assess anxiety.

#### Antidepressant-Like Effect of CBD-Possible Involvement of CB1 Receptors

In recent years, the ECS has attracted interest due to its involvement in the pathophysiology of neuropsychiatric disorders, including depression.^132^ Thus, the modulation of this system could be an exciting tool for their treatment. Accumulating evidence supports the role of CB1 receptors (CB1Rs) in regulating the response to stress, anxiety, and depression.^133^ For example, mutant mice lacking CB1Rs (CB1R knockout, CB1KO mice) displayed anhedonia before control (wild type) mice when exposed to chronic mild stress,^134^ suggesting genetic deletion of CB1R results in an increased susceptibility to the development of an anhedonic state in these animals. Although evidence suggests that CBD has a very low affinity (in the micromolar range) for the CB1Rs, it also has many pharmacological actions and potentiates endocannabinoid neurotransmission by inhibiting the hydrolysis or the reuptake of AEA.^100^ A study by de Morais et al. showed that both CBD and URB597 (the FAAH inhibitor) acute treatments induced antidepressant-like effects in normoglycemic rats, while this phenomenon was not observed in diabetic (DBT) rats.^135^ Interestingly, normoglycemic rats treated acutely with URB597 exhibited a lower frequency of immobility and a higher frequency of swimming and climbing compared to CBD-treated normoglycemic rats.^135^ Conversely, subchronic treatment with CBD, but not with URB597, induced a mild antidepressant-like effect in DBT rats, suggesting CBD has a favorable profile of antidepressant-like activity in DBT rats.

**Figure 6.**
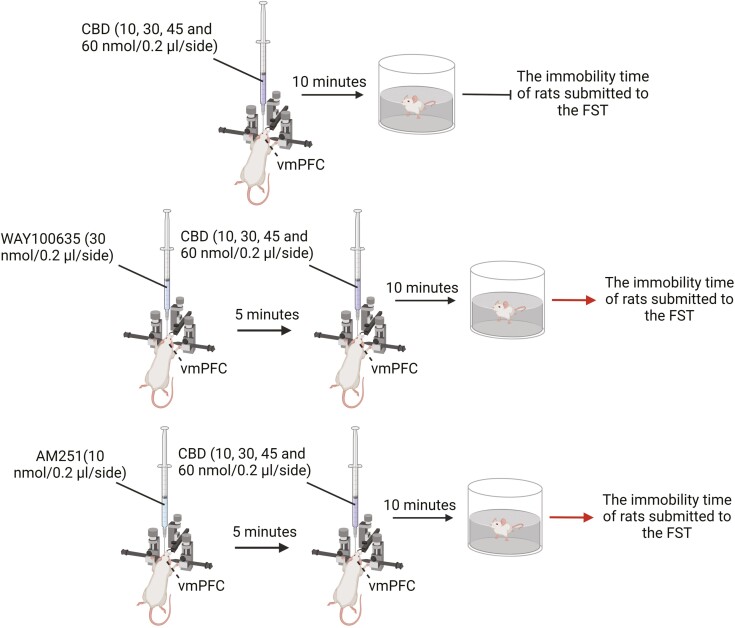
The acute antidepressant-like effect induced by CBD in rat vmPFC is mediated by both CB1 and serotonin receptor subtype 1A (5-HT1A) activation.^107^ Acute administration of CBD (microinjections) into the rat vmPFC at the doses of 10, 30, 45, or 60 nmol reduced the immobility time in the FST compared to the vehicle-treated group. In addition, pre-administration of AM251 (CB1 antagonist, 10 nmol/0.2 μL) or WAY100635 (30 nmol/0.2 μL) prevented CBD-induced antidepressant-like effects. Therefore, these data indicate that the acute antidepressant-like effect induced by CBD in the vmPFC of rats is mediated by both CB1 and 5-HT1A activation. The figure was generated using BioRender (Agreement number: VO27NE5HYF). CBD, cannabidiol; vmPFC, ventromedial prefrontal cortex.

In an important study, Sartim et al. investigated the potential antidepressant effects of CBD in rats.^107^ Acute administration of CBD (microinjections) to the rat vmPFC, either to the PL at doses of 10, 30, or 60 nmol or to the IL subregions at the doses of 45 and 60 nmol, reduced the immobility time in the FST compared to vehicle-treated group.^107^ This finding indicates that both vmPFC subregions are involved in the antidepressant-like effects of CBD after microinjection, albeit with different sensitivity to the drug. Although the exact extent of CBD diffusion from its injection site cannot be accurately assessed, the findings observed here are unlikely to be due to neighboring regions, as no behavioral effects were observed when CBD was injected into regions adjacent to the PL or IL. Furthermore, pretreatment with AM251(CB1 antagonist, 10 nmol/0.2 μL) or WAY100635 (30 nmol/0.2 μL) prevented CBD-induced antidepressant-like effects.^107^ Therefore, these data indicate that the acute antidepressant-like effect induced by CBD in rat vmPFC is mediated by both CB1 and 5-HT1A activation (Figure 6). Given that the serotonergic system of the vmPFC is under the control of local endocannabinoid levels and CB1R activation,^136^ it could be speculated that CBD modulates serotonin release in the vmPFC by indirectly activating CB1Rs. However, a study by Mishima et al. indicated that the neuroprotective effect of CBD may be related to an increase in cerebral blood flow via the serotonergic 5-HT1A receptor but not the CB1R,^137^ suggesting that CBD has a CB1R-independent mechanism, and this hypothesis warrants further investigation.

In line with these findings, a study by Chaves et al. revealed that 2 weeks of CBD treatment was able to induce antidepressant-like effects in DBT rats induced by streptozotocin (STZ) in the modified forced swim test.^138^ STZ-induced diabetes mellitus (DM) provides a very inexpensive and rapid technique that is widely used in experimental animals.^139^ Furthermore, the antidepressant-like effects of CBD in DBT rats (decreased the frequency of immobility and increased the frequency of swimming) are mediated by the activation of 5-HT1A, CB1, and CB2 receptors (CB2Rs), as all these antagonists were able to block this effect of CBD.^138^ These results are consistent with previous studies demonstrating the involvement of CB1R in the antidepressant effects of CBD.^107,124^ Although CBD has a low affinity for the CBR subtypes CB1R and CB2R, CBD acts by inhibiting the FAAH enzyme and consequently, there is an increase in AEA levels, facilitating responses mediated by CBRs. CB2 receptors are known to be preferentially located in the periphery, but also in the central nervous system (CNS).^140^ Accumulating evidence shows that activation of CB2Rs is associated with a decrease in pro-inflammatory cytokines and pro-apoptotic factors.^141^ In addition, numerous in vitro and in vivo studies have confirmed the role of CB2Rs in the management of DM by regulating insulin secretion in pancreatic β-cells.^142,143^ Therefore, it plays a crucial role in the modulatory effect of CBD on glycemic control and also in the CBD-mediated antidepressant-like effect on depression.

In contrast, there are conflicting opinions regarding the activation of CB1R in the antidepressant-like effects of CBD. Austrich-Olivares et al. suggested that low and medium acute doses of CBD (10 and 20 mg/kg) induced antidepressant-like effects in the behavioral tests assessed in CD1 (wild type) mice, while the absence of CB1R did not prevent the antidepressant effects of CBD on coping behavior in the tail suspension test in CB1KO mice.^144^ Based on these results, it is tempting to speculate that the antidepressant effects of CBD may be mediated through other receptors, such as the 5-HT1A receptor described above. An important observation from an in vivo study by Domingos et al.^145^ showed that repeated treatment with CBD (10 mg/kg, daily systemic injections for 7 days) induced antidepressant-like effects via increasing synaptic plasticity markers, including extracellular signal-regulated kinase 1, metabotropic glutamate receptor subtype 5, and synaptophysin levels in the PFC of Flinders Sensitive Line (FSL) rats,^145^ but failed to reverse the reduced CB1 and CB2 levels in FSL rats.^145^ These findings suggest that the antidepressant-like effects of CBD in FSL animals are associated with changes in synaptic plasticity in the PFC, but without increasing the levels of CBRs in this brain region.

Remarkably, 5-HT1A levels were increased in the PFC of the control group (FSL Veh) when compared to Flinders Resistant Line (FRL) Veh, and no difference in 5-HT1A levels was observed between FSL CBD and FSL Veh,^145^ indicating that CBD did not alter this level. In fact, the density of 5-HT1A receptors in FSL rats is significantly different from that in FRL rats,^146^ and FSL rats had a lower density of 5-HT1A receptors but a higher density of 5-HT1B receptors when compared to FRL rats.^146^ Given this difference, we speculate that lower extracellular levels of 5-HT would lead to an upregulation of 5-HT1A receptor sites in the PFC, assuming no change in the receptor affinity.

In summary, these animal data support the involvement of CB1R in CBD-induced antidepressant-like effects (Table 2). Nevertheless, some studies on the role of CB1R in the antidepressant-like effects of CBD remain controversial. These discrepancies may be due to differences in methodological procedures, such as different animal species, strains, behavioral tests used, and the pattern of administration. The exact mechanisms behind this phenomenon need to be further investigated in future studies.

**Table 2.** Several in vivo studies suggest the involvement of CB1R in CBD-induced antidepressant-like effects.

CBD dose	Samples	CB1 antagonist	Mechanism	References
10-60 nmol/side, microinjections	vmPFC of rats	AM251 (10 pmol/side, microinjections)	AM251 blocked CBD-induced antidepressant-like effects	^107^
30 mg/kg, i.p.	Diabetic rats	AM251 (1 mg/kg i.p.)	Antidepressant-like effects of CBD in diabetic rats are mediated by 5HT1A, CB1, and CB2 receptors activation	^138^
20 mg/kg; i.p.	CB1KO mice	SR141716A (2 mg/kg; i.p.)	The lack of CB1R did not modify the antidepressant activity of CBD	^144^
10 mg/kg; i.p.	PFC of FSL rats		CBD effects in FSL animals are associated with changes in synaptic plasticity markers and synaptophysin signaling in the PFC, without increasing the levels of CBRs in this brain region	^145^
15, 30, and 60 nmol, microinjection	The prelimbic division (PrL) of the medial prefrontal cortex (mPFC) in chronic constriction injury (CCI) rats	AM251 (at 200 pmol, microinjection)	AM251 and WAY100635 in the PrL cortex attenuated the antidepressive effect caused by CBD, indicating CBD action into the PrL cortex can be due to the activation of CB1 and 5HT1A receptors	^147^

Abbreviations: CBD, cannabidiol; CBRs, cannabinoid receptors; CB1R, CB1 receptor; FSL, Flinders sensitive line; PFC, prefrontal cortex; vmPFC, ventral medial prefrontal cortex.

#### GPR55 as a Key Target Gene May Mediate Antidepressant-Like Effect of CBD

Although GPR55 has been considered a CBD receptor, it is phylogenetically distinct from CB1 and CB2Rs, as it lacks the classical cannabinoid binding pocket.^148^ GRP55 is widely distributed in the CNS and peripheral tissues and is involved in the regulation of physiological processes such as inflammatory response, pain perception, and emotion regulation.^149,150^ In 2007, Ryberg et al. identified GPR55 as a novel target for CBD and endocannabinoids as well.^151^ CBD acts as a GPR55 antagonist, blocking the pro-excitatory effect of the lipid LPI, an endogenous GPR55 agonist.^152^ Although GPR55 is sensitive to a number of cannabinoids,^153^ the most consistently described agonist is the endogenous lipid, LPI.^152^ LPI drives GPR55-mediated Ca^2+^ flux at neuronal cell bodies and presynaptic hippocampal CA3-CA1 excitatory terminals,^154,155^ while CBD attenuates LPI-mediated presynaptic Ca^2+^ increases and prevents neuronal excitability by reducing glutamate (Glut) exocytosis.^98^

**Figure 7.**
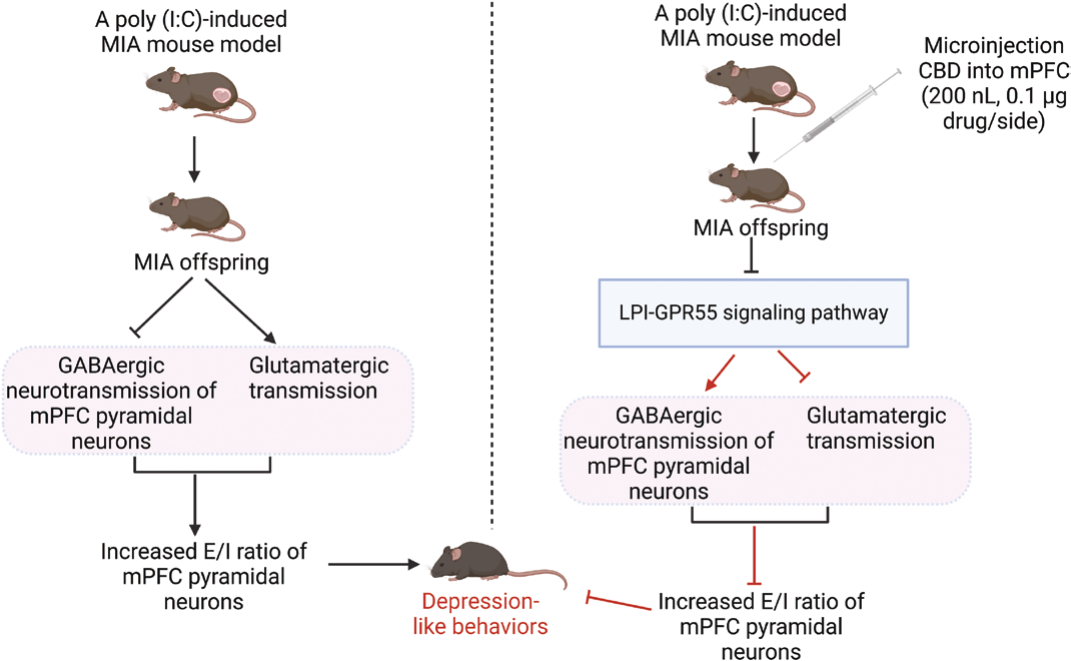
CBD reduces depression-like behaviors in MIA mice via inhibition of the LPI-GPR55 pathway.^109^ LPI, an endogenous GPR55 agonist, activated GPR55 in the mPFC and resulted in healthy animals exhibiting MIA-like phenotypes that were prevented by CBD treatment.^109^ MIA selectively increased excitatory synapses and decreased inhibitory synapses, ultimately leading to an increased excitatory-inhibitory (E/I) ratio of mPFC pyramidal neurons in MIA offspring compared to PBS controls.^109^ In addition, CBD treatment reversed the dysfunction in excitatory and inhibitory neurotransmission and modulated the E/I balance to counter depression-like behavior in MIA mice.^109^ Moreover, a GPR55 antagonist, CID16020046, could mimic the beneficial effects of CBD.^109^ These results indicate that the CBD’s therapeutic effects are mediated via the LPI-GPR55 signaling pathway. The figure was generated using BioRender (Agreement number: ZC27NE5NGZ). CBD, cannabidiol; LPI, lysophosphatidylinositol; MIA, maternal immune activation; mPFC, medial prefrontal cortex.

A recent study by Zhang et al. showed that CBD (30 mg/kg, i.p.) alleviated depression-like behavior in MIA mice by inhibiting the LPI-GPR55 signaling pathway (Figure 7).^109^ They found that MIA selectively increased excitatory synapses and decreased inhibitory synapses, ultimately leading to an increased excitatory-inhibitory (E/I) ratio of mPFC pyramidal neurons in MIA offspring as compared to phosphate buffered saline (PBS) controls.^109^ Furthermore, CBD treatment reversed the dysfunction in excitatory and inhibitory neurotransmission and modulated the E/I balance to counteract depression-like behavior in MIA mice.^109^ These results indicate that the therapeutic effects of CBD are mediated via the LPI-GPR55 signaling pathway. Consistent with this, as first demonstrated by Shen et al. in the spinal nerve ligation (SNL) mice treated with CBD at a dose of 5 mg/kg injected intraperitoneally for 14 consecutive days after surgery,^150^ CBD could effectively alleviate the depressive-like behavior of mice after SNL surgery. Further experiments indicated that the expression level of GPR55 in the spinal cord tissues of the SNL model group was significantly elevated compared to the control group, while CBD treatment effectively reversed the increased level of GPR55 in the spinal cord tissues of SNL mice.^150^ These findings suggest that regulation of GPR55 expression by CBD may contribute to its anti-inflammatory effects in SNL mouse models, thereby exerting its antidepressant effects.

Similarly, in another study, CBD at a dose of 20 mg/kg induced antidepressant-like effects in GPR55KO mice.^144^ In addition, they also chose real-time PCR to measure the relative gene expression of GPR55 in the amygdala (AMY) and hippocampus of wide-type mice and found that GPR55 was increased only at a dose of 30 mg/kg in the AMY, while a significant reduction was observed in the hippocampus at all doses tested (10, 20, 30 mg/kg).^144^ This result further supports the antidepressant-like effects of CBD, which follow an inverted U-shaped curve and are effective at intermediate doses, but not at very low or high doses.^156^

Moreover, CBD also altered the gene expression of GPR55 in a dose- and brain region-dependent manner, suggesting that the antidepressant-like effects of CBD may be due to a multimodal mechanism involving different key targets and brain regions.

In conclusion, the above study provides evidence supporting the potential of CBD in depression through the downregulation of GPR55 expression. Further studies are needed to elucidate the specific mechanisms by which CBD interacts with GPR55 and its downstream signaling pathways.

#### CBD and PPARγ Interaction in Depression

—The discovery of biological targets other than CB1 and CB2 has been pursued in many recent studies, including GPR55, serotonin 5-HT1A, and PPARγ.^157^ There is considerable evidence that some of the significant pharmacological effects of CBD are mediated by its interaction with PPARγ,^158^ suggesting that PPARγ is a key target for CBD. Given the safety and tolerability of CBD in clinical practice, we need to expand our knowledge of the biological targets of CBD. Therefore, we reviewed studies on the interaction of CBD and PPARγ in depression.

PPARs are a group of nuclear transcription factors that includes 3 isoforms: PPARα, PPARβ/δ, and PPARγ.^159^ They are widely distributed throughout the brain and body in various organ systems and play a major regulatory role in energy homeostasis.^160^ Among these, PPARγ is the most extensively studied member of the PPAR family and the most common target for therapeutic intervention. There is ample evidence that the PPARγ system is widespread in the brain, rapidly senses CNS cellular stress, and functions in the CNS in neurons in multiple anti-inflammatory and neuroprotective ways.^161,162^ In addition, PPARγ elevation can positively impact several key pathological processes in depression,^163^ indicating that PPARγ agonists may have significant antidepressant effects.

Mori et al. induced a mouse model of bilateral common carotid artery occlusion (BCCAO) and administered vehicle or CBD (10 mg/kg) 0.5 h before and 3, 24, and 48 h after reperfusion to explore the neuropharmacological mechanisms of CBD action.^164^ BCCAO is a stroke model that can lead to depressive-like behaviors and impairments in cognition and motor function.^165^ CBD significantly reversed an increase in the immobility time of the FST in BCCAO mice compared to BCCAO mice receiving vehicle alone, while pretreatment with GW9662 (a PPARγ receptor antagonist) blocked the antidepressant-like effects of CBD in BCCAO mice.^164^ These findings indicate the involvement of PPARγ in modulating the antidepressant-like effects of CBD in BCCAO mice. Unexpectedly, the GW9662, when administered alone, increased the latency for the first episode of immobility in BCCAO mice.^164^ It has been speculated that the improvements in depressive symptoms are partly mediated by the anti-inflammatory and metabolic regulatory effects of PPARγ.^166^ Therefore, we suggest that the behavioral variability shown by BCCAO mice with GW9662 on FST could be a possibility of a false-positive finding, highlighting the need for further investigation.

Similarly, Esposito et al. have shown that PPARγ promotes hippocampal neurogenesis via PPARγ involvement in rats.^167^ They observed that CBD reduced neuroinflammation sustained by astrocytes through selective activation of PPARγ in vitro cultured astrocytes and in rat hippocampal homogenates, while blockade of PPARγ with GW9662 reversed these effects.^167^ In addition, CBD treatment reversed the massive reduction in neurogenesis in the DG caused by amyloid beta (Aβ) exposure in vehicle-inoculated rats through selective activation of PPARγ, while its effect was almost completely abolished by GW9662.^167^ According to these observations, CBD could promote adult hippocampal neurogenesis by activating PPARγ, further indicating that PPARγ agonists may exert a profound anti-inflammatory and neuroprotective effect.^168^

## LIMITATIONS

CBD is the most abundant nonpsychoactive component of cannabis. It is now clear that CBD interacts with a wide variety of molecular targets in the brain, and its therapeutic potential has been investigated in a number of neuropsychiatric disorders, including depression and anxiety. Although the pharmacological effects of CBD in depression have been extensively investigated by in vitro studies, the mechanisms responsible for its therapeutic potential are still unclear. Here, we review the major biological effects of CBD, focusing on the antidepressant properties of CBD. The antidepressant effects of CBD are currently thought to involve multiple molecular mechanisms of action, including the serotonin 5‐HT1A, CB1 and CB2, GPR55, and PPARγ receptors. Despite this strong evidence, no adequate clinical trial has investigated whether CBD can reduce depressive symptoms in patients. Clinical trials are therefore important to confirm this possibility.

A proposed molecular target for CBD is the TRPV1 receptor (also known as the capsaicin receptor).^104^ Initial observations by Bisogno and colleagues^100^ describing CBD as a TRPV1 receptor agonist were confirmed by De Petrocellis et al.^101^ who also observed rapid desensitization of the TRPV1 channel following CBD application. A recent pivotal study by Gray et al. showed that a significant increase in mean convulsive current (CC50) was observed in TRPV1 knockout mice with CBD treatment (100 mg/kg) compared to the knockout vehicle group, suggesting TRPV1 is a key target involved in the mechanism of anticonvulsant action of CBD.^169^ However, no study to date has investigated the involvement of TRPV1 mechanisms in the antidepressant-like effects of CBD. We recommend that future CBD studies should take these interactions into account.

Some studies suggest the existence of bidirectional interactions between the ECS and the orexin/hypocretin (OX) system.^170^ CBD and OX receptors overlap in several brain areas and have been shown to form heterodimers.^171,172^ In addition to heteromerization, activation of OX receptors induces the synthesis of the endocannabinoid 2-AG,^173^ indicating that the ECS is involved in some physiological functions of OXs. Indeed, functional interactions between the ECS and the OX system have been demonstrated in several behavioral responses, including anhedonia.^170^ It was found that mice treated with the orexin receptor type 2 agonist YNT-185, the CB1R antagonist SR141716A, and the combination of SR141716A and YNT-185 showed less anhedonia after a 24 h restraint stress test (24 h RST) compared to vehicle mice, supporting the dual regulation of CBD and OX in anhedonic behavior induced by prolonged restraint stress.

In addition, reports suggest that the therapeutic properties of CBD may involve epigenetic mechanisms, including DNA methylation, histone modifications, and the regulation of miRNA expression.^97^ Epigenetic mechanisms bridge the genetic and environmental factors that contribute to the pathophysiology of depression. A recent study by Sales et al. found that the antidepressant-like effects of CBD were associated with the modulation of DNA methylation levels and DNA methylation (DNMT) activity in the PFC and hippocampus of mice subjected to the FST.^174^ In addition, CBD (20 mg/kg/i.p.) increased the post-translational modification levels on the histones H3K4Me3, H3K9ac, and H3K27Me3 in the cerebral cortex of rats, thereby reducing depression-like behaviors.^175^ Similarly, the antidepressant effects of CBD in a rat model of depression are associated with changes in miR-16 and miR-135 in the vmPFC.^35^ These findings provide initial clues to the underlying epigenetic mechanisms that may mediate the antidepressant effects of CBD and warrant further investigation in rodent models of depression.

Although it is important to recognize the beneficial effects of CBD, it is even more important to understand the safety and efficacy of CBD. While several high-quality systematic reviews and meta‐analyses of CBD safety have been conducted recently and generally conclude that CBD has a remarkably safe profile,^176,177^ minor side effects are reported, for example, after excluding trials in childhood epilepsy, the only adverse outcome associated with CBD treatment was diarrhea.^177^ Larger, more robust clinical trials are therefore needed to confirm the safety of oral doses of CBD.

## CONCLUSIONS

In summary, there is growing evidence that CBD may be a promising candidate for the treatment of depression. The receptor mechanisms underlying CBD’s effects are very complex and involve in multiple receptors including CB1, CB2, GPR55, 5-HT1A, and PPARγ receptors, as reviewed above. However, its therapeutic use has some limiting factors. CBD has high hydrophobicity and very low water solubility. CBD has also shown exceptionally low oral-gastrointestinal (oral-GI) bioavailability. Furthermore, single acute doses of CBD cause an inverted U-shaped dose-response pattern in human subjects subjected to an experimental model of anxiety,^178^ making the therapeutic window of CBD narrow. Therefore, further preclinical studies and future clinical trials are needed to determine optimal doses.

## Data Availability

All the data extracted from included original articles are available in PubMed or Web of Science. This review was conducted without previous registration, and no protocol document was prepared.
